# Evaluation of a Novel *Aspergillus* Antigen Enzyme-Linked Immunosorbent Assay

**DOI:** 10.1128/JCM.00136-19

**Published:** 2019-06-25

**Authors:** Karl Dichtl, Ulrich Seybold, Steffen Ormanns, Heidi Horns, Johannes Wagener

**Affiliations:** a Max von Pettenkofer-Institut für Hygiene und Medizinische Mikrobiologie, Medizinische Fakultät, LMU München, Munich, Germany; b Sektion Klinische Infektiologie, Medizinische Klinik und Poliklinik IV, Klinikum der Universität, LMU München, Munich, Germany; c Pathologisches Institut der Ludwig-Maximilians-Universität, Medizinische Fakultät, LMU München, Munich, Germany; d Medizinische Klinik und Poliklinik III, Klinikum der Universität, LMU München, Munich, Germany; e Institut für Hygiene und Mikrobiologie, Julius-Maximilians-Universität Würzburg, Würzburg, Germany; f National Reference Center for Invasive Fungal Infections (NRZMyk), Jena, Germany

**Keywords:** *Aspergillus fumigatus*, ELISA, aspergillosis, diagnosis, galactomannan, invasive fungal infections

## Abstract

Invasive aspergillosis (IA) is a life-threatening infection that mainly occurs in immunocompromised patients. Here, we compared the novel *Aspergillus*-specific galactomannoprotein (GP) enzyme-linked immunosorbent assay (ELISA) (Euroimmun Medizinische Labordiagnostika AG) to the established Platelia *Aspergillus* galactomannan (GM) ELISA (Bio-Rad Laboratories) for the detection of IA. A total of 267 serum samples from 45 cases of proven and 4 episodes of probable IA (according to European Organization for Research and Treatment of Cancer/Invasive Fungal Infections Cooperative Group and the National Institute of Allergy and Infectious Diseases Mycoses Study Group [EORTC/MSG] criteria) and 156 sera from patients without evidence of IA were tested. Pearson’s correlation statistics, as well as sensitivity and specificity, were calculated using manufacturer-recommended (GM) or optimized (GP) cutoff levels. Aspergillus fumigatus was found in 88% of culture-positive infections. When we analyzed all 423 serum samples, GM and GP tests correlated strongly (*r* = 0.82, *P* < 0.0001). Among proven IA cases using samples obtained as closely as possible to the day of proven diagnosis, the sensitivity for both tests was 40%. All cases of probable IA (defined by positive GM testing) were also GP positive. Concordant results of the two ELISAs were obtained in 43 of 49 samples (88%). Extending measurements to all sera available in the time frame of 7 days prior to 7 days after the day of proven diagnosis, 47% and 56% of the cases were detected by the GM and GP tests, respectively. Specificity was 99% for GM and 96% for GP testing. For the diagnosis of IA, sensitivity and specificity of the novel GP ELISA are similar to those of the Platelia GM ELISA. The low sensitivities of both tests underline the need for serial testing in patients at risk for IA.

## Abstract (precis)

Invasive aspergillosis (IA) is a life-threatening infection that mainly occurs in immunocompromised patients. Here, we compared the novel *Aspergillus*-specific galactomannoprotein (GP) enzyme-linked immunosorbent assay (ELISA) (Euroimmun Medizinische Labordiagnostika AG) to the established Platelia *Aspergillus* galactomannan (GM) ELISA (Bio-Rad Laboratories) for the detection of IA.

## INTRODUCTION

Invasive aspergillosis (IA) is caused by molds of the genus *Aspergillus* and is characterized by high mortality rates that range from 35% to 90% (1). Immunodeficiency and immunosuppressive therapies, e.g., myeloablative conditioning and hematopoietic stem cell transplantation (HSCT), are major risk factors for IA (2). A key determinant for survival is early treatment, which relies on a timely diagnosis (3). Clinical and radiological signs of IA are often nonspecific, and additional diagnostic tests are required to substantiate diagnosis in almost all clinical settings (4, 5). These include cultivation of the pathogen from infected body sites and analysis of respiratory specimens and blood for fungal biomarkers.

Current biomarkers for IA involve the detection of fungal antigens such as galactomannan (GM) and β-1,3-d-glucan (BDG), as well as the detection of *Aspergillus*-specific DNA (*Aspergillus* PCR) (5). While GM is largely specific for *Aspergillus*, BDG is a panfungal biomarker and cannot discriminate *Aspergillus* infections from other fungal infections, such as Pneumocystis jirovecii pneumonia or candidiasis (6). Most medical centers in Europe therefore employ GM for routine diagnostics and surveillance of patients at risk for IA. Since the launch of the first GM-based *Aspergillus*-specific antigen assay almost 2 decades ago (Platelia *Aspergillus* antigen ELISA; Bio-Rad Laboratories), several attempts have been undertaken to develop alternative tests for the detection of *Aspergillus* antigens. In 2008, Thornton reported the generation of a monoclonal antibody (JF5) that specifically detects an extracellular *Aspergillus*-specific glycoprotein antigen secreted constitutively during the active growth of the mold (7). This antibody was subsequently used to develop lateral flow devices (LFDs) for the analysis of blood and bronchoalveolar lavage (BAL) fluid (7–9). However, LFDs are not compatible with high-throughput testing in routine diagnostic laboratories for surveillance of patient at risk for IA. In addition, robust studies with significant patient cohorts that evaluated the performance of the tests are still scarce (7–10).

In this work, we compared the performance of a new *Aspergillus*-specific antigen ELISA with that of the established Platelia *Aspergillus* antigen ELISA for the detection of IA. This novel JF5-based assay, the galactomannoprotein (GP) ELISA, will receive its CE marking and will be commercially available from Euroimmun Medizinische Labordiagnostika AG in 2019. Our study relies on the characterization of 267 samples of 49 cases of probable (*n* = 4) or proven (*n* = 45) IA according to the European Organization for Research and Treatment of Cancer/Invasive Fungal Infections Cooperative Group and the National Institute of Allergy and Infectious Diseases Mycoses Study Group (EORTC/MSG) Consensus Group and therefore represents, to our knowledge, the largest study evaluating the performance of diagnostic laboratory tests for the detection of IA (5, 11). The results of our study demonstrate similar performance of the two tests with respect to sensitivity and specificity.

## MATERIALS AND METHODS

This study was performed at the Max von Pettenkofer-Institute for Hygiene and Medical Microbiology that hosts the central microbiology laboratory for the University Hospital of Ludwig Maximilians University (LMU) Munich, a 2,000-bed university medical center in Munich, Germany. Between 2007 and 2017, we retrospectively identified 48 patients with episodes of IA. A total of 45 episodes met the EORTC/MSG criteria for a proven IA and 4 met that for a probable IA (11). Two episodes of probable IA occurred in the same patient with a 10-month interval and multiple negative GM tests in between. The day of sampling of the specimen that allowed for the diagnosis of proven IA was defined as “day of proven diagnosis.” Correspondingly, the day of the first GM-positive serum was determined to be the “day of probable diagnosis” in episodes of probable IA. Subsequently, we will refer to the day of proven or probable diagnosis as day 0. In total, 267 sera that were sampled in a period of 6 weeks before and 2 weeks after day 0, stored at −20°C, were available. For a negative-control cohort, we analyzed serum from 156 consecutive patients without suspicion of IA who met the inclusion criteria of being (i) outpatients with the suspicion of borreliosis or (ii) patients with a recent history of HSCT (≤7 days before sampling of the serum).

GM analysis using the Platelia *Aspergillus* antigen ELISA (Bio-Rad Laboratories, Marnes-la-Coquette, France) was performed according to the manufacturer’s instructions upon arrival of the sample (sera of IA cases and of the high-risk control group) or in parallel to GP testing (sera of the negative-control group without suspicion of IA). Indices were rounded to one decimal place with a lower limit of 0.1. Briefly, for the GP assay, 300 μl of serum were mixed with 100 μl of sample buffer, heated in a boiling water bath, and centrifuged at 10,000 × g for 10 min. Supernatant (100 μl per well) was incubated for (i) 1 hour without reagent, (ii) 1 hour with biotin solution, and (iii) 30 min with enzyme conjugate. Each incubation was performed at room temperature on an orbital shaker (275 rounds per minute) with subsequent washing steps (3×) after the indicated incubation periods. Finally, samples were treated with chromogenic substrate 15 min followed by addition of the stop solution. Photometry was performed at 450 nm using a Tecan Sunrise absorbance microplate reader (Tecan Group, Männedorf, Switzerland). Indices (optical density [OD] of sample/OD of cutoff control) of ≥0.4 were considered positive. GP measurements were performed in 2017 and 2018 from frozen samples. Clinical information and reference standard results were not available to the performers and readers of the test.

Statistical analysis was performed using GraphPad Prism 5 (GraphPad Software, La Jolla, CA, USA). Statistical significance was assumed based on an α-level of 0.05.

This retrospective study was reviewed and approved by the ethics committee of our university hospital (Ethikkommission der Medizinischen Fakultät der LMU München) and a waiver of informed consent was granted. Sample processing and data analysis were performed anonymously.

## RESULTS

To characterize and compare the performance of the new *Aspergillus* antigen ELISA, we identified 45 and four cases that meet the EORTC/MSG criteria of proven and probable IA, respectively. In 35 individuals, *Aspergillus* was cultivated from samples obtained from physiologically sterile body sites (78% of proven IA cases, Table 1). Ten episodes of proven invasive fungal infection were characterized by histology only (22% of proven IA cases). Among these cases, we identified six with positive *Aspergillus* cultures in additional specimens. A. fumigatus was by far the most frequently isolated species (88% of culture-positive infections). Other species isolated were Aspergillus flavus and Aspergillus niger (*n* = 4 and *n* = 1, respectively). Demographic characteristics of the 49 cases of IA are depicted in Table 2. The most common underlying conditions were hematologic malignancies (39%) and history of solid organ transplantation (29%). Nine individuals (18%) required intensive care treatment due to other medical indications. In two cases, predisposing factors and/or pathogenesis of IA remained unclear.

**TABLE 1:** Characteristics of infection

Characteristic	Cases	
	*n*	%
EORTC/MSG category		
Proven IA	45	92
By culture	35	71
By histology only^*a*^	10	20
Probable IA^*b*^	4	8
Focus of IA		
Lung	29	59
Intraabdominal	6	12
Circulatory system	5	10
Central nervous system	3	6
Bones and joints	2	4
Urogenital tract	2	4
Orbita	1	2
Skin	1	2
Species isolated^*c*^		
Proven cause of infection		
A. fumigatus	30	61
A. flavus	4	8
A. niger	1	2
Cultivation only from nonsterile body site		
A. fumigatus	6	12

a In six of the ten histology positive cases, A. fumigatus was cultivated from additional samples.

b Two episodes of probable IA were diagnosed in the same patient.

c Cases with *Aspergillus* isolates cultivated from specimen that allowed for categorization as proven invasive aspergillosis (IA) are listed in the subsection “proven cause of infection.” The subsection “cultivation only from nonsterile body site” lists isolates obtained from nonsterile body sites of patients whose diagnosis of proven IA is based on histology only.

**TABLE 2:** Demographic characteristics and underlying conditions of invasive aspergillosis cases

Characteristic or condition	Cases	
	*n*	%
Demographic characteristic		
All cases of proven/probable IA	49	100
Female sex	19	39
Median age	60	
Interquartile range	42–67	
Underlying condition		
Hematologic malignancy	19	39
History of HSCT^*a*^	12	24
Solid organ transplantation	14	29
Lung	8	16
Heart	3	6
Liver	2	4
Kidney	1	2
Intensive care treatment	9	18
Following surgery	7	14
Solid organ malignancy	3	6
Immunosuppressive therapy^*b*^	1	2
DiGeorge syndrome	1	2
Undiagnosed comorbidity	2	4

a HSCT, hematopoietic stem cell transplantation.

b The category “immunosuppressive therapy” does not include patients with a history of transplantation.

**FIG 1:**
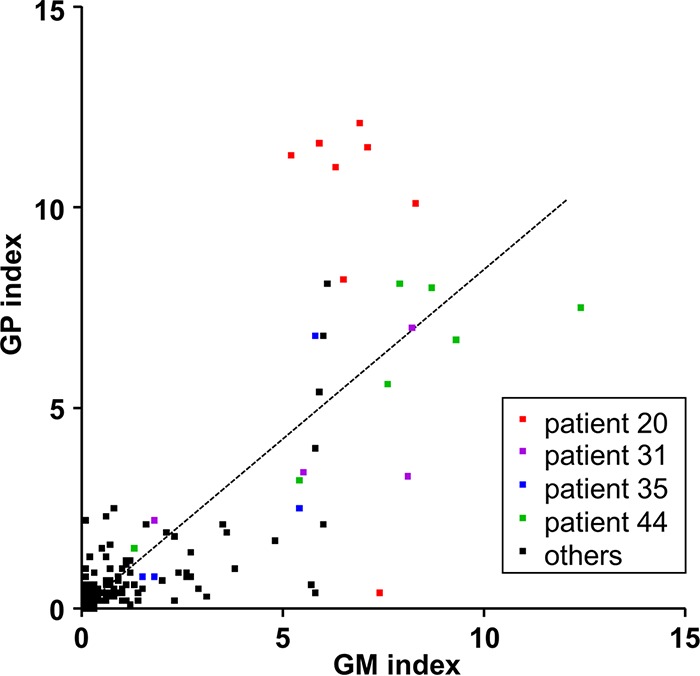
Correlation of the optical density (OD) indices determined for individual serum samples of patients with probable or proven invasive aspergillosis. 423 serum samples were analyzed. Measurements of selected individuals (patients 20, 31, 35, and 44) with multiple highly positive indices are indicated with colors. Patients 20, 31, and 35 suffered from IA with invasion of blood vessels. Patient 44 suffered from pulmonary IA. *x* axis, OD index of the sample measured with the galactomannan (GM) antigen ELISA; *y* axis, OD index of the sample measured with the galactomannoprotein (GP) ELISA; the dotted line indicates linear regression analysis.

**FIG 2:**
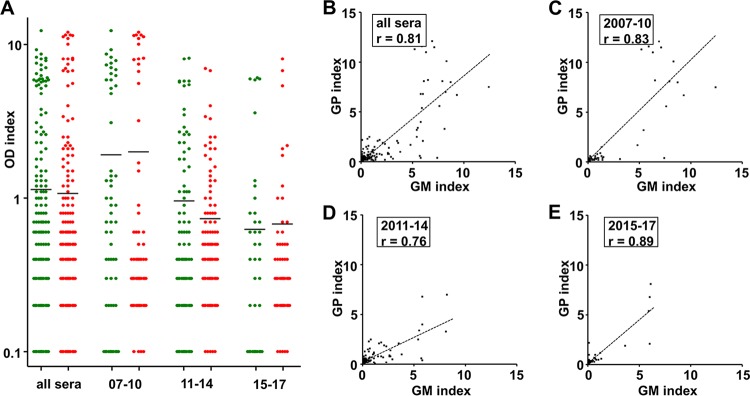
Antigen stability. A total of 267 sera from 49 cases of proven/probable IA were sampled between 2007 and 2017. Measurement results (indices of optical density [OD]) of the galactomannan (GM, green; analyzed upon arrival of the sample in the diagnostic laboratory) and the galactomannoprotein (GP, red; analyzed from frozen samples in 2017 and 2018) ELISA are plotted for different sampling periods (A). The subset “all sera” contains all specimen sampled between 2007 and 2017. Black bars mark the median of results. Measurement results of the two ELISAs of all sera (B) and the subsets of different sampling periods (C to E) were correlated. *r*, Pearson’s correlation.

First, we aimed to determine the quantitative agreement between the optical density (OD) indices of the different ELISAs by Pearson’s correlation. Analyzing all 423 samples, we observed a strong correlation of *r* = 0.82 (*P* < 0.0001) between the measurement results of both tests (Fig. 1). A total of 95 and 127 specimens tested positive by the GM and the GP assay, respectively (Table 3). A total of 81 samples tested positive by both assays. Twenty-seven GM-positive and 17 GP-positive measurements yielded highly positive results (>5.0). Most of these measurements belonged to a limited number of individuals with notable clinical characteristics, namely, three cases of intravascular infection accounted for more than half of the highly positive results of each test (Fig. 1). Importantly, a comparison of the range and distribution of GP and GM OD indices of the 267 sera of IA cases did not provide evidence of antigen degradation during storage (Fig. 2A). The correlation of measurement results was high in all temporal subgroups (Fig. 2B to E).

**TABLE 3:** Concordance of measurement results of all included samples^*a*^

Assay and result	No. (%) of samples with GM result	
	Positive	Negative
GP		
Positive	81 (19)	45 (11)
Negative	14 (3)	283 (67)

a Cutoffs of 0.5 and 0.4 were used for GM and GP antigen testing, respectively.

**FIG 3:**
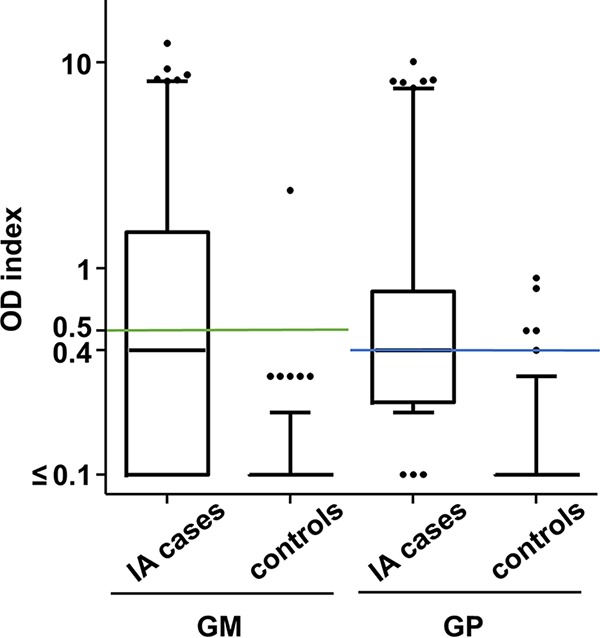
Distribution of the measurement results of the two *Aspergillus* antigen ELISAs. Serum samples of IA patients and those of an *Aspergillus*-negative control group were analyzed with the galactomannan (GM; A) and galactomannoprotein (GP; B) ELISAs. The optical density (OD) indices are depicted as box plots. Whiskers mark 5th and 95th percentiles. The green and blue lines indicate the manufacturer-recommended (GM) or optimized (GP) cutoff levels, respectively, for the individual tests (0.5 for the GM ELISA and 0.4 for the GP ELISA).

Next, we analyzed sera of an *Aspergillus*-negative control group to determine the specificity of the tests. This *Aspergillus*-negative control group included 156 sera in total, 79 sera from outpatients with the suspicion of borreliosis and 77 sera from patients at high risk of IA due to a recent history of HSCT (≤7 days before sampling of the serum), but no cultural or histologic evidence for IA. The distribution of the ELISA results is depicted in Fig. 3 Interestingly, one sample from a control patient was highly positive in both tests.

**FIG 4:**
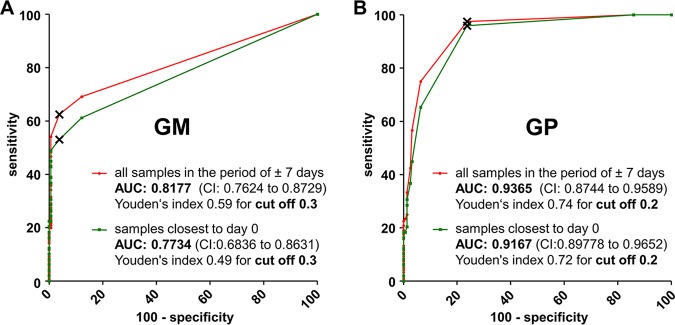
Receiver operating characteristic (ROC) curves for the two *Aspergillus* antigen ELISAs. Based on the measurement results of the indicated sample groups, ROC curves for the galactomannan (GM) antigen ELISA (A) and the galactomannoprotein (GP) ELISA (B) were plotted in the depicted graphs. Data points with the maximum Youden’s index for the individual curves are labeled (×). AUC, area under the curve; CI, confidence interval.

For each assay, two receiver operating characteristic (ROC) curves were plotted, which relied on different data sets for the case group (Fig. 4). The first analysis is based on the measurement of one serum per patient that was dated closest to day 0 for each of the 49 IA cases. The second curve was calculated using all available sera of all 49 IA cases that were sampled in a time slot of ±7 days from day 0 (*n* = 120). The distribution of the measurement results is depicted in Fig. 3 For both assays, the area under the curve (AUC) was larger when the ±7 days case group was applied (Fig. 4). However, both approaches resulted in a maximum Youden’s index for a GM cutoff 0.3 (0.49 and 0.59, respectively) and for a GP cutoff 0.2 (0.72 and 0.74, respectively). When applying the ROC analysis-based cutoffs, specificities for the GM and the GP ELISA were 96% and 76%, respectively.

Since *Aspergillus* serology is used for screening and surveillance of patients at risk for IA, a high specificity of the assay is essential. The manufacturer of the GM assay therefore suggests a cutoff 0.5, which results in one false-positive case in our control group and a specificity of 99%. In favor of a higher specificity, we applied an adapted (optimized) cutoff 0.4 for the GP ELISA, which resulted in five false-positive cases (including the sample that also tested positive in the GM ELISA) and a specificity of 97%. OD indices of the false positives were 0.4, 0.5, 0.5, 0.8, and 0.9. Interestingly, when the GP ELISA was repeated with the five positive samples, all but one samples yielded negative results. The repetitive positive sample (GP OD index 0.9) was identical with the false-positive sample in the GM assay (GM OD index, 2.4).

Based on the recommended and optimized cutoffs (0.5 for GM and 0.4 for GP analysis), we calculated the sensitivities of the tests (Table 4). To this end, we focused on the serum for each IA case that was obtained closest to day 0. The mean time period between sampling of the serum and day 0 was less than 2 days (median, 0 days); 60% of the serum samples dated from a period of ±24 h from day 0, and all sera were obtained in a time frame of ±1 week. Both assays were positive in 18 of 45 proven IA cases (40%). All four cases of probable IA (defined by GM seropositivity) were positive by the GP ELISA. Concordant test results of the two ELISAs were obtained in 43 of 49 samples. Each test identified three cases in which the competitor failed. Notably, in most of these cases positive and/or negative results tended to be close to the cutoff. Combining both tests increased the sensitivity for the detection of the 45 proven IA cases to 47%.

**TABLE 4:** Sensitivities and specificities

Sensitivity and specificity data	Test	
	GM	GP
Sensitivity (%)^*a*^		
All cases	45	45
Cases of proven IA only	40	40
Hematologic patients (proven IA)	53	40
Nonhematologic patients (proven IA)	33	40
Serologic diagnosis of IA (%)^*b*^		
Serum sampled ±7 days from day 0	51	59
Cases of proven IA only	47	56
Hematologic patients (proven IA)	60	53
Nonhematologic patients (proven IA)	40	57
Serum sampled −6 to +1 weeks from day 0	55	71
Cases of proven IA only	51	69
Hematologic patients (proven IA)	67	67
Nonhematologic patients (proven IA)	43	70
Specificity (%)		
All control sera	99	97
High-risk patients^*c*^	100	96
Suspicion of borreliosis	99	97

a For calculation of sensitivities, only the serum sample closest to day 0 of each case was considered.

b Serologic diagnosis of IA is defined by at least one seropositive sample in the indicated period.

c Sera of high-risk patients were sampled ≤7 days following hematopoietic stem cell transplantation.

**FIG 5:**
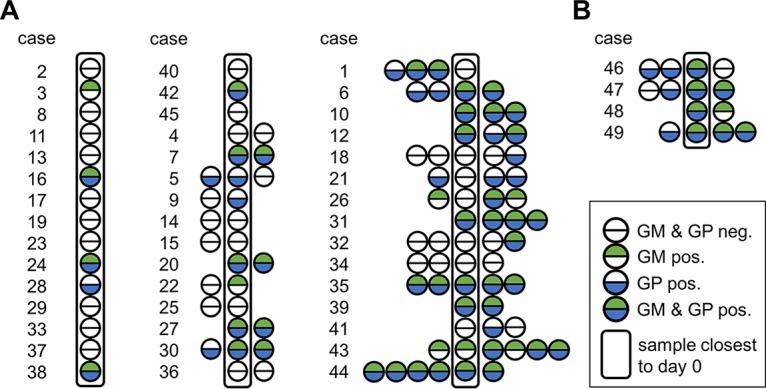
Results of antigen testing in all sera of cases of proven or probable IA obtained in the period of ±7 days from the day of proven or probable diagnosis. A total of 120 serum samples of patients diagnosed with proven (A) or probable (B) IA were analyzed with the galactomannan (GM) and the galactomannoprotein (GP) ELISAs. Each circle represents a serum sample (depicted in chronologic order). Sera sampled closest to the day of proven (A) or probable (B) diagnosis are enclosed in frames. Green and blue semicircles indicate positive test results for GM and GP testing, respectively. Empty semicircles indicate negative test results.

Additional sera from the time frame of ±7 days from day 0 were available in 27 cases of proven IA. By including these samples, 16 (59%) and 20 (74%) of the 27 cases were detected by the GM and GP tests, respectively. Similarly, when all sera of all 45 proven IA cases in the time frame of ±7 days from day 0 were included, GM and GP seropositivity was detected in 47% and 56% of the cases, respectively (Table 4, Fig. 5).

**FIG 6:**
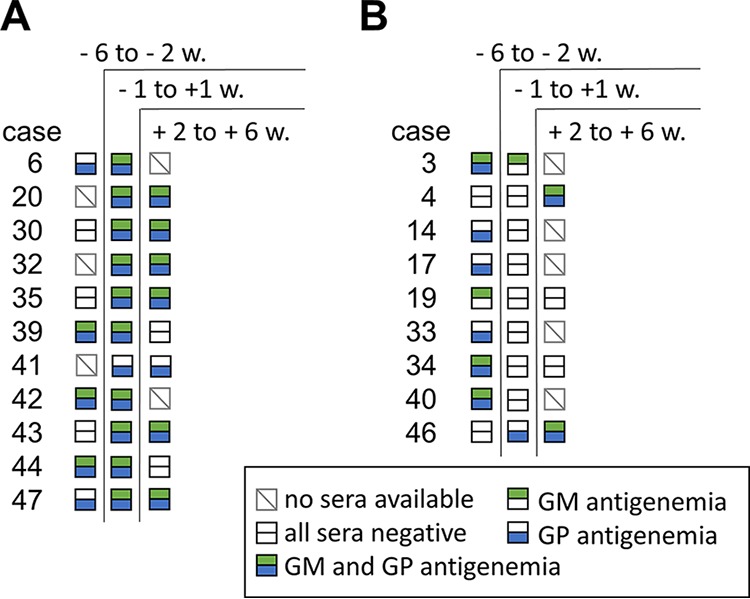
Results of antigen testing in all sera of cases of proven or probable IA during the time course of infection. A total of 267 samples of patients diagnosed with proven or probable IA were analyzed with the galactomannan (GM) and the galactomannoprotein (GP) ELISAs. Samples were obtained in the time intervals of −6 to −2, −1 to +1, and +2 to +6 weeks from day of proven or probable diagnosis, respectively. In 11 cases of IA, seropositivity of at least one test was detected in two or three different time intervals (A). In 9 cases of IA, at least one test yielded positive results in the preceding or succeeding time interval but not in the period of ±1 week (B). Squares represent the three time intervals for each individual case. Green and blue symbols indicate presence of positive test results of the GM and the GP ELISA, respectively, in the respective time interval. Empty symbols indicate negative test results. Gray squares with a backslash (\) indicate that no serum samples were obtained in the respective time interval. w, weeks.

Importantly, based on the inclusion criteria, the day of proven or probable diagnosis (day 0) may not correlate with the day of clinical diagnosis or suspicion of an invasive mold infection. It is possible and likely that many cases had already been clinically diagnosed and treated days or weeks before day 0. Sera obtained in the period of −6 to −2 weeks before day 0 were available for 23 proven IA patients. When testing these samples, we found 7 and 11 cases to be GM and GP positive, respectively (Fig. 6). In contrast, 3 and 6 of these cases were seronegative in the time slot of ±7 days from day 0. If test results of all sera in the time frame of −6 to +1 week from day 0 were included in the analysis (number of sera per case ranged from one to 14), 23 and 31 of 45 patients (51% and 69%) suffering from proven IA had periods of galactomannanemia or galactomannoproteinemia, respectively (Table 4).

The GM ELISA had a better performance in patients with underlying hematologic disease than in nonhematologic patients (sensitivity of 53% versus 33%, Table 4). This was not observed upon GP testing (40% sensitivity in both subgroups). Focusing on nonhematologic patients, the GP ELISA detected more episodes of IA in comparison to GM testing in the subset of sera sampled closest to day 0 of each case (40% versus 33%), in the subset of sera sampled in the period of ±7 days from day 0 (57% versus 40%), and in the subset of sera sampled in the time frame of −6 to +1 week from day 0 (70% versus 43%).

## DISCUSSION

Both the GM and the GP ELISA are based on monoclonal antibodies with high specificity for *Aspergillus* antigens. The rat IgM antibody EB-A2, which is applied in the Platelia GM ELISA, binds to the cell wall polysaccharide galactomannan (12, 13). The IgG3 antibody JF5 was demonstrated to bind to a protein epitope localized in the hyphal cell wall (7). This antigen has an *N*-glycosylated component that remains to be characterized in more detail (7). The protein-carbohydrate complex detected by the JF5 antibody was named galactomannoprotein by the manufacturer of the GP ELISA. The EB-A2 antibody used in the GM ELISA was reported to cross-react with clinically important fungi, such as Cryptococcus neoformans, Candida spp., Talaromyces (formerly *Penicillium*) *marneffei*, and Fusarium solani (14–17). To date, no cross-reactivity was reported for JF5-derived tests. More specifically, culture supernatants of the species mentioned above were not detected as positives in a JF5-based LFD (7).

Previous studies evaluated the sensitivity of the JF5-based LFD in point-of-care settings. For several reasons, e.g., small study populations and uncertainty about the version of the LFD under investigation, reliable data concerning the performance of the LFD are scarce. Studies that included between one and eight cases of proven IA reported sensitivities ranging from 33% to 100% (8–10, 18, 19). Notably, testing of serum resulted in lower sensitivities (33% to 73%) than testing of BAL fluid (18, 19). However, the very different study designs impair a direct comparison of the tests.

The latest generation of the LFD was CE marked in 2018. The novel JF5-based GP ELISA will be CE marked in 2019 and will be the first commercially available *Aspergillus* antigen ELISA since the launch of the Platelia kit almost 20 years ago. One aim of our study was to identify an optimal cutoff for the GP assay. To this end, we characterized sera of 156 patients without suspicion of IA and of four episodes with probable IA (two episodes were in the same patient) and 45 patients with proven IA. ROC curve analysis resulted in a maximum Youden’s index for a GP cutoff 0.2. Applying this cutoff results in a remarkably high sensitivity of 96% but in a low specificity of only 76%. A major purpose of *Aspergillus* antigen testing is screening in high-risk patients, which requires high specificity (4, 5). We therefore propose a cutoff 0.4 for the GP test, which in our study resulted in a specificity of 97%. Importantly, retesting of the false-positive serum samples of the GP assay, a procedure that is recommended by the manufacturer of the GM assay in the case of initial positive results, yielded negative results except for one serum that was also positive in the GM assay. The overall sensitivity of the GP, as well as that for the GM ELISA, was 40% in all sera sampled closest to day 0.

In previous studies, a significantly higher sensitivity was reported for the GM assay (approximately 70 to 85%) (20). One possible explanation could be the inclusion of patients based on the EORTC/MSG histopathologic definitions of proven invasive infection which may suffer from non-*Aspergillus* mold infections (11) (Table 1). However, in most of these cases, additional evidence allowed for a clinical diagnosis of IA. Six cases were culture positive for A. fumigatus in respiratory specimens, and four patients were seropositive in at least one GM test. In one case, neither additional culture-positive nor seropositive results were available. This suggests that the low sensitivity observed in our study is related to other factors, such as changing patient characteristics in present hospital settings. Most of the previous studies were primarily based on high-risk patient cohorts, with a focus on hematologic malignancies. In contrast, the patient cohort analyzed in the present study (i) included a significant number of patients without hematologic malignancies and (ii) several of the patients with hematologic malignancy in our study most likely received antifungal chemoprophylaxis with posaconazole (beginning of chemoprophylaxis in the university medical center, 2009; treatment data for individual patients were not available due to medical data protection). These two characteristics, i.e., IA patients without hematologic malignancies and posaconazole prophylaxis, have been repeatedly reported to negatively affect the sensitivity of the GM assay (21–23). Notably, the GP ELISA does not suffer from impaired sensitivity in nonhematologic patients but was found to yield comparable sensitivities independent of the patients’ underlying disease.

Current guidelines recommend serial screening of serum samples of patients at risk for IA for *Aspergillus* antigen (4, 5). We therefore evaluated the performance of the tests by including multiple serum samples obtained during the course of infection (−6 to + 1 weeks from day 0). Seropositivity was detected in 51% and 69% of cases by GM and GP testing, respectively. This finding suggests a higher per case sensitivity of the GP compared to GM ELISA. However, the evidence of this statistical analysis must be interpreted with caution because of the heterogeneity and poor comparability of the individual cases.

Besides serial screening of serum samples, combined testing for GM and other biomarkers, e.g., BDG or *Aspergillus*-specific DNA, was recommended to increase sensitivity (4, 5). Combining both ELISAs resulted in an improved the sensitivity (40% versus 47%; based on the cohort of proven IA cases) without reducing the specificity. However, the increase in sensitivity is moderate due to a high concordance of the two tests, namely, 86% of all GM-positive sera were also tested positive for GP. It is possible that combining non-GP- and non-GM-based tests, e.g., for BDG or *Aspergillus*-specific DNA, with the tests evaluated in the present study could be more advantageous.

In summary, our data suggest that (i) sensitivity and specificity of the novel GP ELISA are very similar to that of the Platelia GM ELISA and (ii) the low overall sensitivities of the *Aspergillus* antigen ELISAs reinforce the need for serial testing in patients at risk for IA.
